# The genetic puzzle of FAP: exploring novel diagnostic approaches for *APC*/*MUTYH-*negative case

**DOI:** 10.1186/s13053-025-00318-7

**Published:** 2025-11-07

**Authors:** Natalia Grot, Marek Kazimierczyk, Marcin Szuman, Marta Kaczmarek-Ryś, Alicja Kryszczyńska, Iga Dziechciowska, Monika Knaur, Andrzej Hnatyszyn, Szymon Hryhorowicz, Andrzej Pławski

**Affiliations:** 1 Polish Academy of Sciences, Institute of Human Genetics, Strzeszyńska 32, Poznań, 60-479 Poland; 2 Independent Public Health Care Centre in Nowa Sól, Multispecialty Hospital, Nowa Sól, 67-100 Poland; 3 Department of General and Endocrine Surgery and Gastroenterological Oncology, Poznań University of Medical Sciences, Przybyszewskiego 49, Poznań, 60- 355 Poland

**Keywords:** *APC*, Colorectal cancer, FAP, *MUTYH*, Polyposis

## Abstract

Multiple polyposis syndromes include Familial adenomatous polyposis (FAP), Peutz-Jeghers syndrome (PJS), Juvenile polyposis syndrome (JPS), PTEN hamartoma tumor syndrome (PHTS), MUTYH-associated polyposis (MAP), NTHL1-associated polyposis (NAP), Polymerase proofreading-associated polyposis (PPAP), and MBD4-associated polyposis. Common to these syndromes is the presence of polyps in the large intestine and very high risk of developing colorectal cancer (CRC), which can reach up to 100% in the case of FAP. The development of FAP is associated with pathogenic variants of the *APC* gene. However, pathogenic variants are not always detected in patients with FAP, which poses a significant clinical challenge for both patients and their families, who may be at increased risk for developing the disease. A second strong predisposition to CRC is MAP, characterized by biallelic pathogenic variants in the *MUTYH* gene, with a phenotype similar to FAP. This mini review focuses on potential approaches to improve the diagnosis of patients in whom pathogenic variants in the *APC* and *MUTYH* genes are not detected by routine testing.

## State of the art

**Fig. 1:**
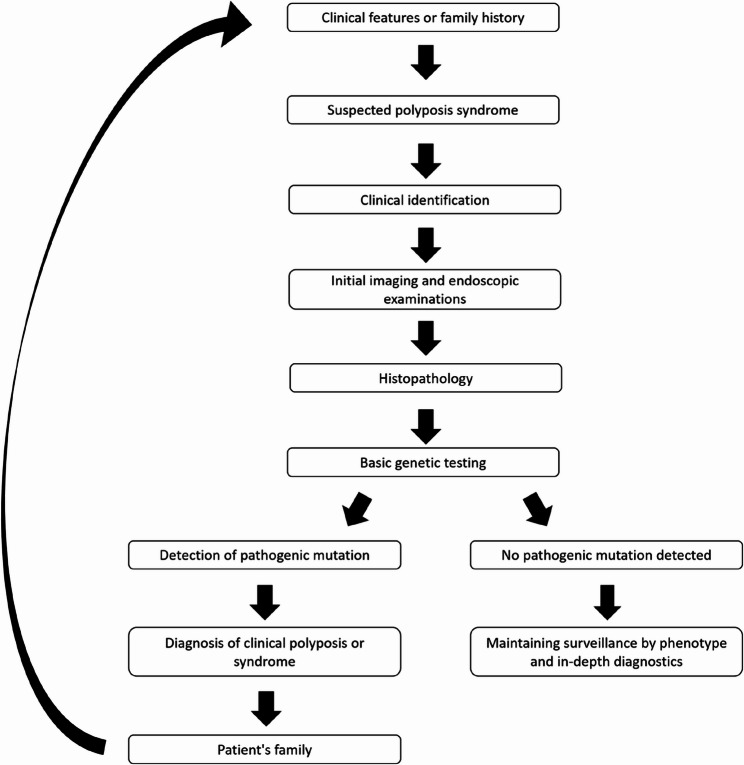
Diagnostic procedure in cases of intestinal polyposis [34–40]

Multiple polyposis syndromes, characterized by multiple colorectal polyps, are a group of diseases encompassing Familial adenomatous polyposis (FAP), Peutz-Jeghers syndrome (PJS), Juvenile polyposis syndrome (JPS), PTEN hamartoma tumor syndrome (PHTS), MUTYH-associated polyposis (MAP), NTHL1-associated polyposis (NAP), Polymerase proofreading-associated polyposis (PPAP), and MBD4-associated polyposis. The syndromes are caused by inherited predisposing variants in specific genes which are responsible for a high risk of developing colorectal cancer (CRC) [1] reaching up to 100% in FAP patients [2]. It should be noted, however, that some of these syndromes – such as PJS or PHTS – also carry a significant risk of extracolonic cancers like pancreatic cancer, female breast cancer, gastric, ovarian, uterine and lung cancers [1, 3], which may be the predominant concern in affected families. Moreover, FAP itself is linked not only to CRC but also the duodenal, small bowel, thyroid, and pancreatic cancers may be observed [4]. Familial adenomatous polyposis (FAP; MIM# 175100) is clinically defined by the presence of numerous colorectal adenomatous polyps, typically starting developing during late childhood and early adolescence. Diagnosis is confirmed by identifying a pathogenic germline variant in the *APC* gene. Notably, phenotypic expression can vary significantly even among individuals with the same *APC* variant, including within the same family [5, 6]. Clinically, FAP is recognized as an autosomal dominant condition predisposing to CRC, characterized by the development of numerous colorectal polyps and associated extracolonic manifestations such as desmoid tumors, osteomas, and other neoplasms. However, an identifiable mutation in the *APC* gene is not found in all cases. Reported detection rates have varied widely – older studies, based on conventional methods, showed 35–80% [7–13], while recent reanalyses using advanced bioinformatics techniques have exceeded 90% in some cohorts [14–20]. Given ongoing improvements in methods and interpretation, the true proportion of genetically unresolved cases remains uncertain. In this mini review, we focus on such cases to explore alternative genetic and non-genetic mechanisms contributing to polyposis. Alterations of the *APC* gene predominantly involve small insertions or deletions, with hotspots at codons 1309 and 1061 [21]. The vast majority of pathogenic variants (> 90%) in the *APC* gene lead to truncation of the protein product [22]. While most mutations are inherited in an autosomal dominant manner, around 25% arise de novo [2]. The course of FAP is associated to the pathogenic variants’s location within the *APC* gene. It should be noted, however, that clinical expression may vary even in individuals with the same pathogenic variant, including within the same family. Although the position of the mutation provides useful prognostic information, the variation in disease course even in carriers of the same mutation should be taken into consideration in genetic counseling [23]. Attenuated familial adenomatous polyposis (AFAP), a less aggressive FAP variant, is associated with specific *APC* pathogenic variants predominantly located upstream of codon 157, downstream of codon 1595, or within the alternatively spliced site of exon 9. Compared to FAP, AFAP is characterized by < 100 adenomatous polyps, a later onset of CRC and a reduced lifetime risk of CRC [24]. However, recent studies challenge this assumption. For instance, a matched cohort study found no statistically significant difference in hazard ratios (HRs) for CRC between AFAP and classical FAP patients (HR = 1.48, *p* = 0.0646) [25]. In our study, untreated AFAP patients with a mutation at the 5` end of the *APC* gene died of cancer in the 3rd decade of life [26]. Phenotypically similar to the AFAP is MUTYH*-*associated polyposis (MAP; MIM# 608456), resulting from biallelic pathogenic variants in the *MUTYH* gene [27, 28]. These variants have been observed in 8% of patients with polyposis in whom mutations in the *APC* gene could not be detected. The most common pathogenic variants in *MUTYH* are Y165C and G382D, accounting for approximately 80% of MAP cases [29, 30]. In 2015, researchers identified a recessively inherited biallelic nonsense mutation c.244 C > T (p.Q82*) in the *NTHL1* gene [31], which disrupts the base excision repair (BER) pathway [32]. This has been linked to a recently described multi-cancer predisposition known as NAP [33]. The search for causative factors for the occurrence of adenomatous polyps in the absence of pathogenic variants in the *APC* and *MUTYH* genes is extremely challenging (Fig. 1).

Clinical features or family history: Diagnosis begins with symptoms such as GI bleeding, iron-deficiency anemia, bowel habit changes, or abdominal pain. Alternatively, a positive family history – especially early-onset colorectal or related cancers – raises suspicion. Clinical red flags include ≥ 10 cumulative adenomatous polyps, multiple hamartomatous/serrated polyps, and extraintestinal features like congenital hypertrophy of the retinal pigment epithelium (CHRPE), mucocutaneous pigmentation, macrocephaly, or desmoid tumors. Suspected polyposis syndrome: These signs prompt suspicion of a hereditary polyposis syndrome and warrant targeted diagnostic evaluation; Clinical identification: Includes a three-generation pedigree and physical examination. Phenotypic clues (e.g. CHRPE for FAP, pigmentation for PJS, or dermatologic/endocrine signs for PTEN syndromes) help guide syndrome-specific testing. Initial imaging and endoscopic examinations: Colonoscopy remains key to assessing number, type, and distribution of polyps. Based on suspicion, additional tests include upper gastrointestinal endoscopy, magnetic resonance enterography/capsule endoscopy, thyroid or abdominal ultrasound, or ophthalmological exams. Histopathology: Resected polyps are analyzed for type (adenomatous, hamartomatous, serrated, mixed) and for dysplasia or malignancy. Histology narrows syndrome suspicion (e.g., hamartomas → JPS or PJS; adenomas → FAP or MAP). Basic genetic testing: Genetic counseling and NGS-based multigene panel testing covering *APC*, *MUTYH*, *SMAD4*, *BMPR1A*, *STK11*, *PTEN*, *POLE*, and *POLD1*. Depending on clinical suspicion, analysis may include mosaicism, copy number variations (CNVs), and non-coding variants. Detection of pathogenic mutation: A molecular diagnosis confirms the syndrome (e.g., FAP via *APC* mutation). This enables personalized management and cascade testing for relatives. Diagnosis of clinical polyposis or syndrome: If no variant is found but phenotype is compelling, diagnosis of clinical polyposis is made. These patients are managed as high risk despite molecular uncertainty. Patient’s family: Cascade testing for first-degree relatives is recommended. Mutation carriers begin surveillance; mutation-negative but high-risk relatives may still require monitoring. No pathogenic mutation detected: Further steps may include testing tumor tissue (mosaicism), evaluating promoter/intronic regions, or advanced sequencing. Ensure sensitivity and CNV coverage were adequate. Maintaining surveillance by phenotype and in-depth diagnostics: Patients who fulfill clinical criteria for polyposis but lack a molecular diagnosis should still undergo regular surveillance based on phenotype. Colonoscopy is typically performed every 1–2 years, with additional screening based on syndrome-specific risks. Genetic data should be re-evaluated periodically as new disease-causing genes or testing methods become available. Research enrollment may also be considered in unresolved cases.

## Insufficient standard *APC* testing - new methods of FAP stage diagnosing

The presence of patients with an FAP phenotype, in whom standard genetic tests fail to detect mutations in the *APC* gene, highlights the need to broaden molecular diagnostic approaches. Analysis of the available data suggests several directions for further investigation.

Already collected and well-characterized samples can be reanalyzed using the latest highthroughput techniques, which provide deeper insights into the molecular basis of the disease. A reanalysis of 31 families out of 231 registered in the Danish Polyposis Registry revealed that some *APC*-negative patients in standard genetic tests carry mosaic mutations in *APC*, which are undetectable on blood DNA but present in polyps (7% of all cases). The combined use of Whole-genome sequencing (WGS), and mosaicism analysis increased the diagnostic success rate to 60% in the genetically unresolved FAP cases [14].

While WGS may contribute to the detection of mosaicism, its sensitivity is limited by typical read depth. Therefore, when mosaicism is suspected, analysis should ideally be performed on DNA extracted from adenomas or tumor tissue, using high-depth targeted sequencing or digital PCR to improve detection of low variant allele fractions.

The causes of FAP are also being investigated for other predisposing mutations, not just those ascending in *APC*. WGS and Whole-exome sequencing (WES) led to the identification of predisposing mutations in novel candidate genes, such as *NTHL1*, *MSH3*, *POLE*, and *POLD1*, suggesting alternative mechanisms of polyposis development [41]. In 2016, a WES study in patients with colorectal adenomatous polyposis identified predisposing mutations in *DSC2*, *PIEZO1*, *ZSWIM7* [42], and *MSH3* [43]. In a manner analogous to the findings regarding *MSH3*, Olkinuora et al. (2019) reported a biallelic truncating variant in *MLH3* (c.3563 C > G, p.Ser1188*) in the Finnish population, which is linked to both classical and attenuated forms of FAP [44]. Furthermore, based on Sanger sequencing analysis of 53 Indian families with FAP, biallelic pathogenic variants in *MUTYH* were identified in four individuals from two families. In the same study, when no pathogenic variants in *APC* or *MUTYH* were detected, sequencing of the entire coding region of *NTHL1* and the exonuclease domain of *POLD1* (exons 6–13) and *POLE* (exons 9–14) was performed. Unfortunately, no pathogenic variants were found in these regions [45]. In a study of Australian patients, WES, mosaicism analysis in *APC*, and CNV analysis were performed, leading to the identification of 16 cases with mutations in DNA repair and inflammation-related genes. Interestingly, despite the extensive whole exome analysis, 21 cases remained without an identified cause [46].

Modern techniques enable a broader diagnosis of FAP, revealing cases with pathogenic variants in genes associated with related polyposis syndromes, such as MAP (*MUTYH*), NAP (*NTHL1*), or PPAP (*POLE/POLD1*). Therefore, current clinical guidelines recommend that patients with suspected FAP undergo multigene panel testing as a first-line diagnostic approach. This should include not only *APC* and *MUTYH*, but also other relevant genes such as *POLD1*, *NTHL1*, *MSH3*, and *AXIN2*. If panel testing fails to identify a pathogenic variant, further steps – such as investigating mosaicism in adenomas, structural variants, or non-coding changes – should be considered.

Importantly, the clinical phenotype of familial adenomatous polyposis (FAP) does not always arise from pathogenic mutations in the well-established predisposition genes *APC* or *MUTYH*. Comprehensive multigene panel testing plays a crucial role in differentiating classic FAP from phenotypically similar but genetically distinct polyposis syndromes, enabling more accurate diagnosis and guiding appropriate surveillance and management strategies. It is important to note that no single gene has been identified that accounts for a significant proportion of individuals presenting with a clinical FAP phenotype in the absence of *APC* or *MUTYH* mutations.

### Deep analysis

#### Of the *APC* gene sequence

It is known that the absence of a functional allele is not only associated with pathogenic variants that truncate its protein product. Studies indicate the effect of deep intron pathogenic variants on alternative gene splicing through the activation of donor and acceptor sites.

Spier et al. demonstrated the presence of pathogenic alterations in the non-coding parts of the *APC* gene, resulting in the incorporation of so-called pseudo-exons into the gene transcript [47]. Nevertheless, this study showed the presence of such a pathogenic variant in only 6% of patients. In the same group of patients, two hotspot pathogenic variants were defined, the first in intron 4 (c.532-941G > A) and the second in intron 10 (c.1048 + 731 C > T). Several other insertional pathogenic variants of the pseudoexon (c.646-1806T > G in intron 6, c.1408 + 729 A > G in intron 11 and c.1408 + 731 C > T in intron 11) have also been detected in Finnish patients [48]. In 2023, Bozsik A. et al. discovered the intronic alteration c.531 + 1482 A > G in the *APC* gene, which was classified as a new pathogenic variant causing FAP [49]. These studies demonstrated the importance of intron changes in the *APC* gene, previously thought to be insignificant in disease determination. In other cases, combined DNA and RNA sequencing revealed deep intron pathogenic variants in APCs, including c.933 + 829 A > G (causing inclusion of 73 nucleotides from intron 8); c.532–934 A > T (including 167 nucleotides from intron 4) and c.730–494 C > G (causing abnormal splicing in intron 6) leading to abnormal splicing and pseudo-exon formation in 12–22% of mRNAs [50]. Splicing errors were also observed in the case of the synonymous APCΔ486s (Tyr486Tyr) pathogenic variant, which, despite not altering the amino acid sequence, can lead to the skipping of exon 12 [51].

#### Of the genes from the *APC* protein complex

*CTNNB1* activating pathogenic variants S45F and T44A were found in three of five colorectal tumors without *APC* gene alterations [52]. Studies have shown that in about half of sporadic neoplasms lacking *APC* pathogenic variants, tumor formation was associated to pathogenic variants in the NH2-terminal regulatory domain of the *CTNNB1* gene [53, 54]. The *AXIN2* gene has also been investigated in several studies in patients with polyposis or colorectal cancer, however, the identified variants have not been found to be pathogenic [55, 56]. *AXIN2* is currently considered a candidate gene with limited evidence for association with colorectal polyposis, as noted in GenCC (HGNC:904) and OMIM (OMIM:604025), particularly in the context of oligodontia-colorectal cancer syndrome.

#### Of structural variants

Despite the extensive application of panel-based and exome sequencing, a subset of patients with a clinical FAP diagnosis remain without detectable pathogenic variants. In some of these cases, the underlying cause involves large-scale genomic rearrangements or mobile element insertions that are not captured by conventional diagnostic approaches.

A substantial proportion of patients with clinical features of FAP remain genetically undiagnosed after standard testing such as gene panels, exome sequencing, and CNV analysis. The case study by Baumann et al. (2025) provides a compelling example of how long-read genome and RNA sequencing can uncover previously undetectable pathogenic variants in such unresolved cases.

The study focused on a five-generation family with a clear FAP phenotype. Extensive routine diagnostics failed to identify a pathogenic *APC* variant. Using long-read sequencing (PacBio), the authors identified a 6.1 kb full-length LINE-1 retrotransposon inserted into intron 7 of the *APC* gene. While this insertion was partially suggested by short-read WGS, its full sequence and pathogenic nature could only be resolved through long-read technology. Complementary RNA analysis revealed aberrant splicing, including the creation of a pseudoexon containing a premature stop codon, which triggered nonsense-mediated decay and led to functional inactivation of one *APC* allele.

This study demonstrates that retrotransposon-mediated structural variants – especially LINE-1 insertions – may be an underrecognized cause of hereditary conditions like FAP. The findings underscore the value of incorporating long-read sequencing and transcript-level validation into the diagnostic pipeline, particularly in genetically unresolved cases [57].

WGS has enabled detection of non-coding structural alterations, including promoter deletions and enhancer disruptions. However, its sensitivity to structural variants depends heavily on sequencing depth and appropriate bioinformatic pipelines. Therefore, the integration of complementary techniques remains crucial for identifying rare or cryptic causes of FAP.

#### Of non-coding regulatory elements

The high percentage of patients without detectable *APC* pathogenic variants suggests a more complex mechanism underlying FAP. Consequently, the search for biomarkers and novel molecular mechanisms has increasingly focused on non-coding RNAs (ncRNAs), particularly microRNAs (miRNAs) and long non-coding RNAs (lncRNAs), which play key regulatory roles.

In a study conducted on six FAP patients in Japan, circulating miRNAs in the blood were analyzed. Compared to seven healthy donors, miR-143-3p, miR-183-5p, and miR-885-5p were found to be significantly upregulated. Among these, miR-143-3p was identified as the most promising biomarker for FAP [58]. miRNAs have also been used to differentiate sporadic desmoid tumors (DTs) from FAP-associated DTs, where specific miRNA expression patterns can help identify FAP-DT cases, which has critical implications for diagnosis and treatment decisions [59, 60]. The pathogenic mechanism of *APC* pathogenic variants primarily involves deregulation of the WNT/β-catenin pathway. In recent years, several miRNAs have been identified that modulate key proteins within this pathway, affecting the progression of FAP. For instance, miR-155-5p is significantly downregulated in FAP, leading to increased expression of *AXIN*1 and *TCF4* and prolonged activation of the WNT pathway [61]. Another regulatory mechanism involves the modulation of miRNAs by lncRNAs through the ceRNA (competitive endogenous RNA) network. The LncRNA acts as a sponge for miR-185-5p, causing deregulation of CCND2, a key cell cycle regulator in FAP cells [62]. A similar mechanism has been observed in FAP cases without detectable *APC* pathogenic variants. In these patients, lncRNA MIA-RAB4B regulates miR-24, leading to the upregulation of the oncogenes *PIM2* and *TAOK1*, thereby contributing to disease progression [63]. Although most studies focus on non-coding sequence alterations, such as intronic splicing defects or disrupted regulatory RNAs, it is important to acknowledge that in rare cases, the FAP phenotype may result from non-sequence-based mechanisms. These may include epigenetic silencing of the *APC* promoter, deregulation of post-transcriptional gene expression by miRNAs [61–63], or the influence of genetic modifiers and environmental factors. While not routinely assessed, these mechanisms could underlie polyposis in patients without identifiable pathogenic variants.

## Conclusions

Some cases of polyposis remain without a clear explanation. Studies on larger populations using newer methods indicate that mutations in *APC* and *MUTYH* continue to be a major cause. The study of non-canonical cases remains valid and shows how complicated and important good diagnostic tests are. Rapid development of sequencing technologies, along with decreasing costs, now allows in-depth analysis of non-coding regions in predisposition genes. However, in routine clinical settings, where cost remains a major limitation, sequencing is often restricted to the coding sequence of the *APC* gene. Expanding molecular diagnostics to include deep intronic and regulatory variants could greatly improve the identification of pathogenic variants in genetically unresolved cases. Although FAP is associated with the 5q21 locus, the lack of detectable coding pathogenic variants in many patients suggests that pathogenic alterations may be located in non-coding regions of *APC*. Several recent studies have highlighted the importance of intronic pathogenic variants, which can lead to aberrant splicing, pseudoexon formation, and loss of gene function. Therefore, WGS and RNA analysis should be considered for patients with a clinical FAP diagnosis but without detectable pathogenic variants in standard tests. Another important factor to consider is the *MUTYH* gene, particularly in cases similar to AFAP. Given the phenotypic overlap between AFAP and MAP, patients with unexplained polyposis should undergo biallelic *MUTYH* pathogenic variant screening. The exclusion of pathogenic variants in this gene is crucial for accurate diagnosis and clinical management. While predisposing variants in genes such as *NTHL1*, *MSH3*, *POLE*, *POLD1*, *DSC2*, and *PIEZO1* have been associated with colorectal polyposis, they do not fully mimic the classical FAP phenotype. Instead, these variants may define distinct polyposis syndromes, which should be recognized as separate clinical entities rather than alternative causes of FAP. Recent studies suggest a regulatory role of ncRNAs, particularly miRNAs and lncRNAs, in FAP pathogenesis. Altered miRNA expression patterns have been observed in FAP patients, including those without detectable *APC* pathogenic variants, suggesting that post-transcriptional regulatory mechanisms may play a role in disease progression. The identification of specific ncRNA biomarkers may improve early diagnosis and provide novel therapeutic targets. In conclusion, unraveling the missing heritability of FAP requires a comprehensive diagnostic approach that includes WGS, RNA sequencing, and functional studies of novel variants. Future research should focus on refining molecular classification criteria, developing novel diagnostic tools, and exploring epigenetic and transcriptomic mechanisms underlying unexplained FAP cases. Expanding routine molecular diagnostics beyond coding sequences will be a key step toward more accurate diagnosis and personalized management of polyposis syndromes.

## Data Availability

No datasets were generated or analysed during the current study.

## Abbreviations

AFAP: Attenuated familial adenomatous polyposis
BER: base excision repair
ceRNA: competitive endogenous RNA
CHRPE: congenital hypertrophy of the retinal pigment epithelium
CNV: copy number variation
CRC: colorectal cancer
DT: desmoid tumor
FAP: Familial adenomatous polyposis
HR: hazard ratio
JPS: Juvenile polyposis syndrome
lncRNA: long non–coding RNA
MAP: MUTYH–associated polyposis
miRNA: microRNA
NAP: NTHL1–associated polyposis
ncRNA: non–coding RNA
PHTS: PTEN hamartoma tumor syndrome
PJS: Peutz–Jeghers syndrome
PPAP: Polymerase proofreading–associated polyposis
WES: Whole–exome sequencing
WGS: Whole–genome sequencing
